# Supplementary material for: Investigating factors driving shifts in subtype dominance within H5Nx clade 2.3.4.4b high pathogenicity avian influenza viruses
Source: J Gen Virol. 2025 Sep 1;106(9):002150. doi: 10.1099/jgv.0.002150 (PMC12401460; doi:10.1099/jgv.0.002150)
Supplement: Uncited Supplementary Material 1. [file jgv-106-02150-s001.pdf]

Table S1

Table S1. Viruses used in this study.

Abbreviation	Virus strain name	Description	EURL genotype	Subtype	GISAID accession number
H5N1-W1	A/mute swan/England/234255/2020	Wave 1 H5N1	C	H5N1	EPI_ISL_766876
H5N1-W2	A/chicken/Scotland/054477/2021	Wave 2 H5N1	AB	H5N1	EPI_ISL_9012696
H5N8-W1	A/chicken/England/030786/2020	Wave 1 H5N8	A	H5N8	EPI_ISL_17212363
H5N8-W2	A/mute swan/England/298902/2021	Wave 2 H5N8	A	H5N8	EPI_ISL_13369742
SB-H5N1-W1	A/mute swan/England/234255/2020	Wave 1 H5N1 with MBCS replaced with SBCS	C	H5N1	EPI_ISL_766876*
SB-H5N8-W1	A/chicken/England/030786/2020	Wave 1 H5N8 with MBCS replaced with SBCS	A	H5N8	EPI_ISL_17212363*

**wild type* sequence; multi-basic cleavage site (MBCS); single basic cleave site (SBCS).

Table S2

Table S2. Total counts of each clinical sign observed following infection with H5N1-21 or H5N8-21 with or without prior exposure.

Clinical Sign	Individual clinical sign severity score (1-7)	Group					
		H5N1-W2	H5N8-W2	N1/N1	N1/N8	N8/N8	N8/N1
Found dead	12	0	0	0	0	0	0
Euthanised	11	0	3	0	2	6	1
Changes in huddling	1	0	7	3	2	5	4
Eyes closed	1	0	1	1	0	0	1
Conjunctivitis	1-2*	4	1	10	4	20	36
Changes in body position	1	0	12	13	18	13	11
Oedema	2-3	0	0	0	0	0	0
Cyanosis of extremities	2-3*	0	0	0	0	0	0
Lethargy	1	2	21	10	9	15	5
Lack of engagement with enrichment	1	0	22	9	5	5	2
Oronasal discharge	2	0	1	0	0	1	0
Diarrhoea	2-3*	0	13	4	7	8	16
Perceived weight reduction	1-3*	1	1	0	0	1	0
Loss of balance	2	0	2	0	0	6	2
Tremors	2-3*	1	7	0	8	6	5
Torticollis	4	0	1	0	0	0	0
Seizure	7	0	1	0	2	1	0
Paralysis	7	0	0	0	0	0	1
**Total**		**8**	**90**	**50**	**55**	**81**	**83**

* Range in score depends on severity of the clinical sign

Table S3

		H5N8-W2	H5N1-W2	H5N1-W1	H5N8-W1
PB2	**H5N8-W2**	100.00	98.16	98.68	99.47
	**H5N1-W2**	98.16	100.00	98.94	97.89
	**H5N1-W1**	98.68	98.94	100.00	98.41
	**H5N8-W1**	99.47	97.89	98.41	100.00
PB1	**H5N8-W2**	100.00	98.55	98.21	99.74
	**H5N1-W2**	98.55	100.00	99.04	98.28
	**H5N1-W1**	98.21	99.04	100.00	98.21
	**H5N8-W1**	99.74	98.28	98.21	100.00
PA	**H5N8-W2**	100.00	98.46	98.10	99.72
	**H5N1-W2**	98.46	100.00	99.12	98.74
	**H5N1-W1**	98.10	99.12	100.00	98.39
	**H5N8-W1**	99.72	98.74	98.39	100.00
HA	**H5N8-W2**	100.00	99.29	99.46	99.65
	**H5N1-W2**	99.29	100.00	99.46	99.65
	**H5N1-W1**	99.46	99.46	100.00	99.82
	**H5N8-W1**	99.65	99.65	99.82	100.00
NP	**H5N8-W2**	100.00	97.99	97.93	99.00
	**H5N1-W2**	97.99	100.00	98.96	98.39
	**H5N1-W1**	97.93	98.96	100.00	98.96
	**H5N8-W1**	99.00	98.39	98.96	100.00
NA	**H5N8-W2**	100.00	30.94	30.29	97.95
	**H5N1-W2**	30.94	100.00	96.64	30.02
	**H5N1-W1**	30.29	96.64	100.00	29.32
	**H5N8-W1**	97.95	30.02	29.32	100.00
MP	**H5N8-W2**	100.00	98.11	99.02	99.37
	**H5N1-W2**	98.11	100.00	98.37	98.74
	**H5N1-W1**	99.02	98.37	100.00	99.67
	**H5N8-W1**	99.37	98.74	99.67	100.00
NS	**H5N8-W2**	100.00	86.10	88.00	98.84
	**H5N1-W2**	86.10	100.00	97.63	86.43
	**H5N1-W1**	88.00	97.63	100.00	88.35
	**H5N8-W1**	98.84	86.43	88.35	100.00

H5N1-W1 (A/mute swan/England/234255/2020), EPI_ISL_766876; H5N8-W1 (A/chicken/England/030786/2020), EPI_ISL_17212363; H5N1-W2 (A/chicken/Scotland/054477/2021), EPI_ISL_9012696; H5N8-W2 (A/mute swan/England/298902/2021), EPI_ISL_13369742.

Table S3. Amino acid (aa) differences between different H5Nx HPAIVs using concatenated protein sequences. Colour shading represents the size of the values within table S3; values are shaded continuously from red (smallest value, 29.32) through to green (largest value, 100).

Fig. S1

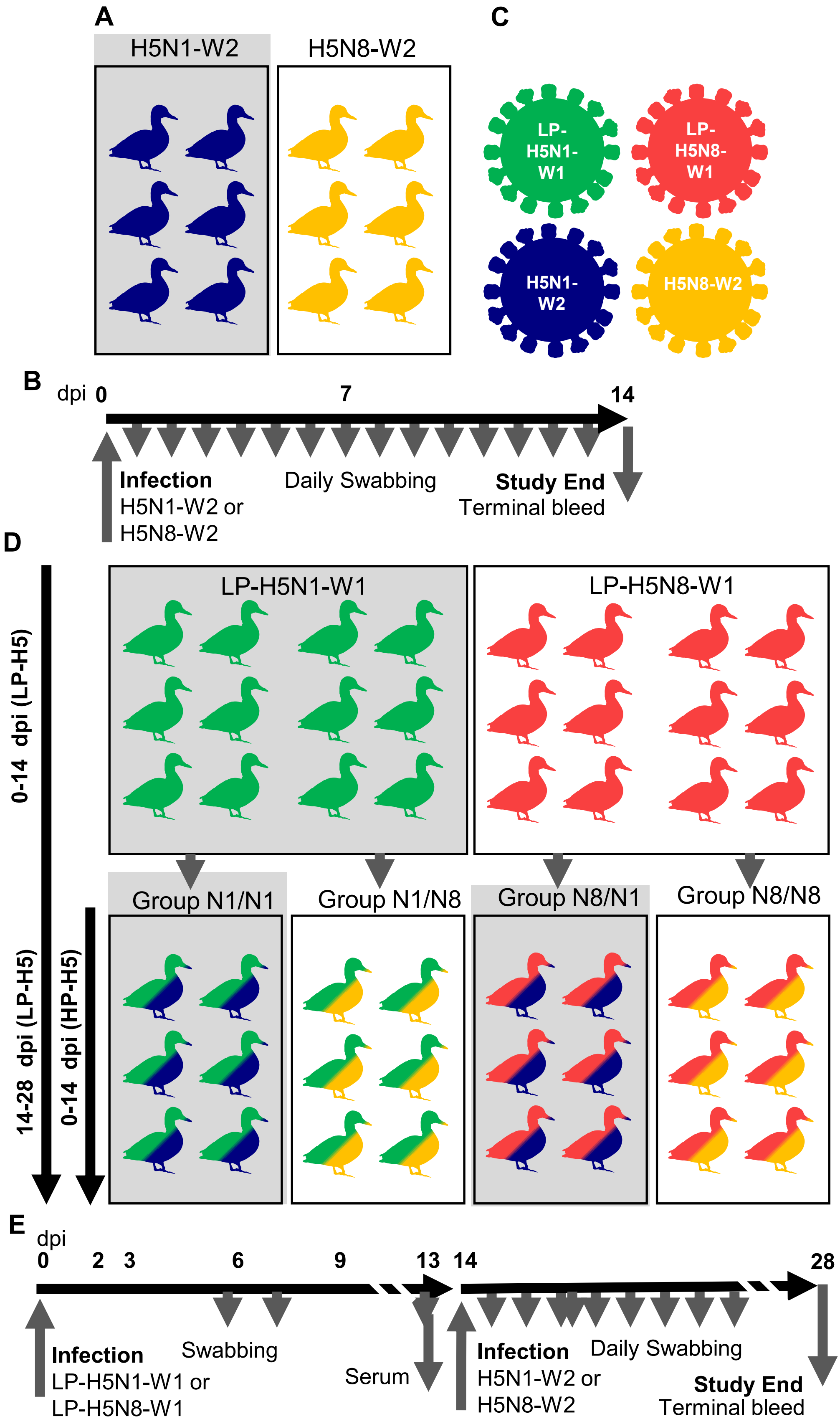

Fig. S1. H5N1-W2 and H5N8-W2 *in vivo* experimental designs.

A and B. Schematic diagram of the experimental design used to assess variation in fitness between H5N1-W2 and H5N8-W2 in naive ducks. **C**. Viruses used in this study. **D and E**. Schematic diagram of the experimental design used to assess infection outcomes from HPAIV H5N1-W2 or H5N8-W2 in ducks which had previously been inoculated with a homologous (N1/N1 and N8/N8) or heterologous (N1/N8 and N8/N1) subtype of de-engineered H5N1-W1 or H5N8-W1 AIVs.

Fig. S2

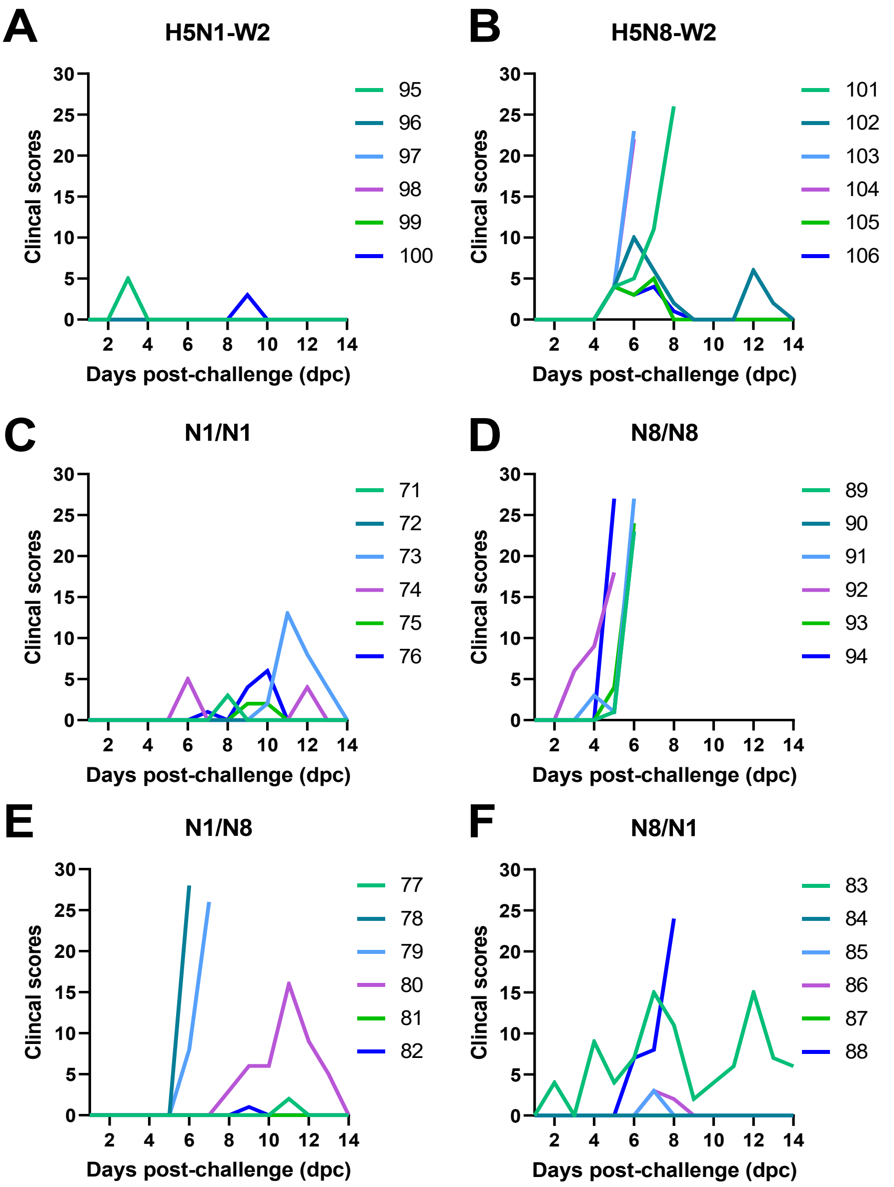

Fig. S2. Clinical scores following infection with H5N1-W2 or H5N8-W2 with or without prior exposure to LPAIV.

Individual clinical scores exhibited per timepoint following infection with HPAIV H5N1-W2 (**A**) or H5N8-W2 (**B**) without prior exposure, or in birds which had previously been exposed to a homologous (N1/N1 (**C**) and N8/N8 (**D**)), or heterologous (N1/N8 (**E**) and N8/N1 (**F**)) subtype of de-engineered H5N1-W1 or H5N8-W1 AIVs. Ducks were scored for severity of clinical scores a minimum of twice daily during the study. Each duck's total daily score has been plotted for 1-14dpc.

Fig. S3

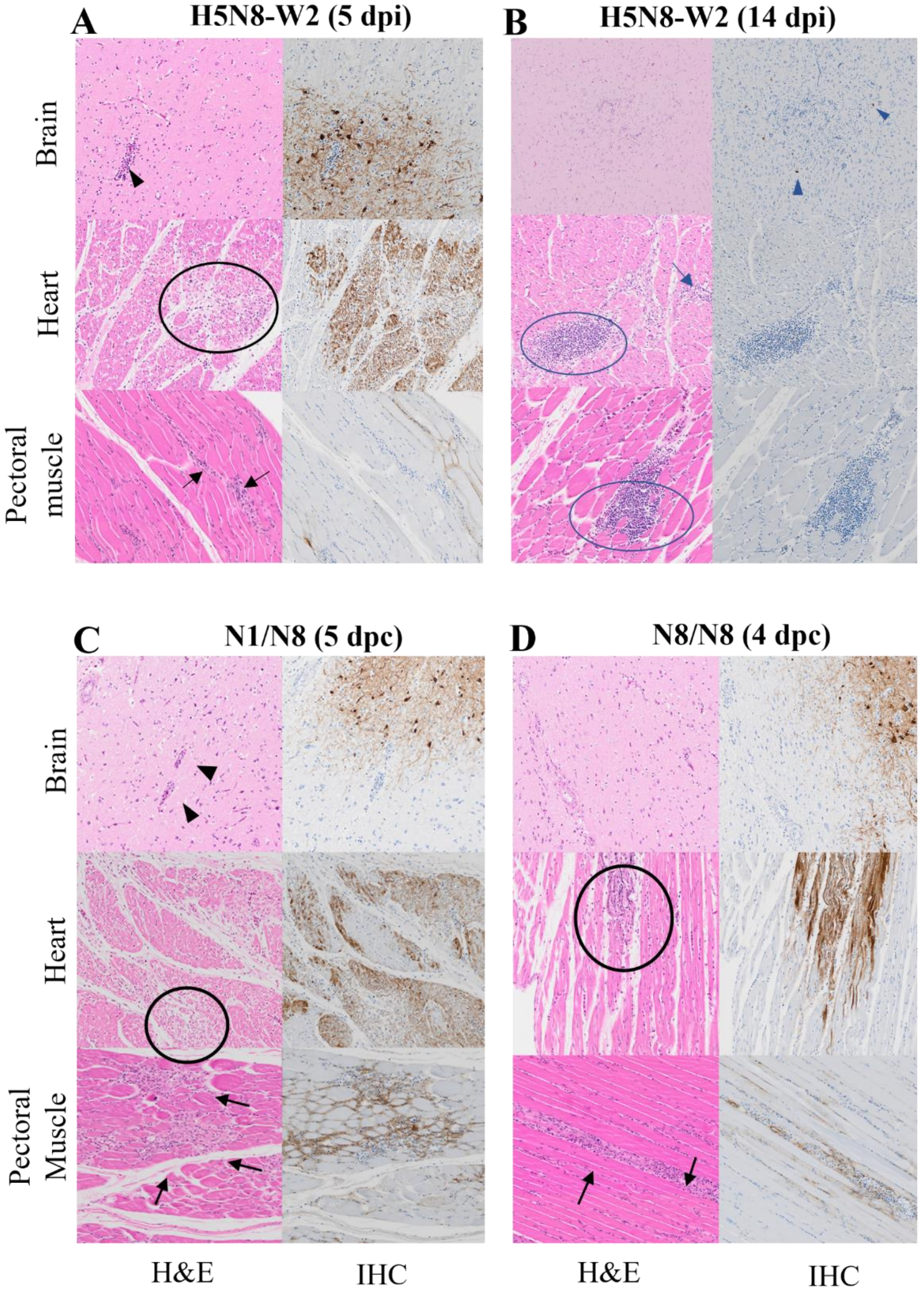

Fig. S3. Representative histopathology (H&E) and immunohistochemistry (using an anti-influenza A nucleoprotein antibody staining brown) demonstrating comparison of changes in heart, muscle, and brain from naïve ducks infected with H5N8-W2 at 5 dpi (**A**) and 14 dpi (**B**), and those inoculated with SB-H5N1-W1 followed by challenge with H5N8-W2 (N1/N8) at 5dpc (**C**), with SB-H5N8-W1, followed by challenge with H5N8-W2 (N8/N8) at 4 dpc (**D**). Acute lesions seen at 4-5 dpi (**A**) or dpc (**C** and **D**) were multifocal acute nonsuppurative encephalitis with mild lymphohistiocytic perivascular cuffing (arrowheads) and abundant immunolabelling in neurons in the brain, myocardial degeneration/necrosis (circles), and myositis, with viral antigen seen in cardiomyocytes in the heart and myocytes in pectoral muscle. Chronic changes at 14dpi (**B**) show minimal multifocal gliosis in the brain and multifocal lymphoid hyperplasia and mild lymphocytic inflammation in heart and muscle, with minimal viral antigen in neurons and macrophages representing viral clearance. Limited lesions were observed in N8/N1 ducks; no samples from N1/N1 were available. Magnification: x20. Days post infection (dpi), days post challenge (dpc).

Fig. S4

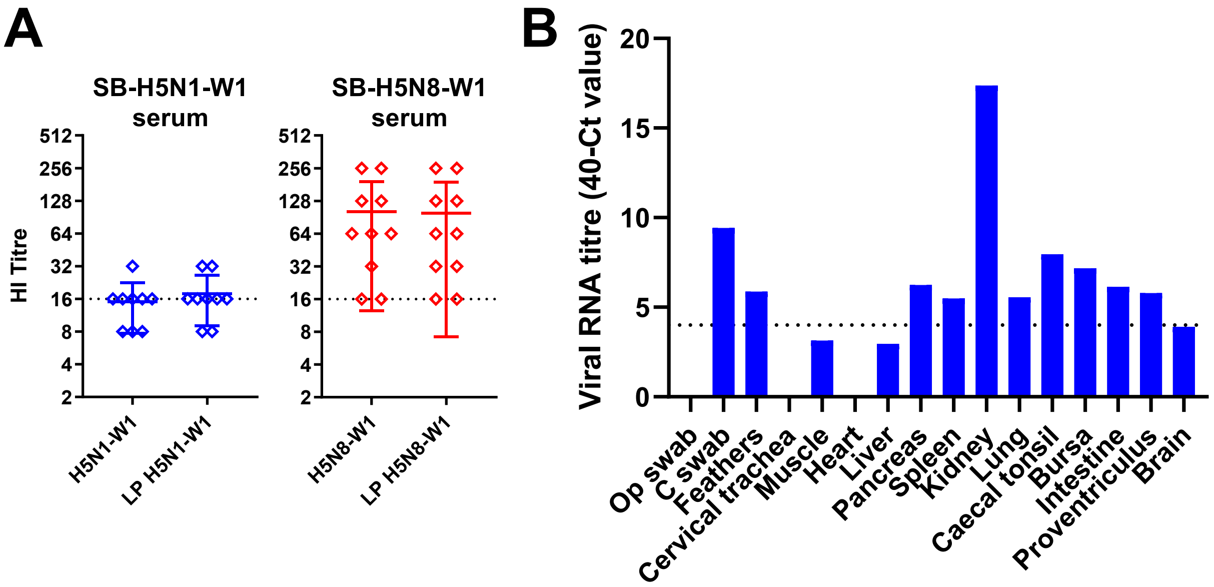

C

	-9	-8	-7	-6	-5	-4	-3	-2	-1	0	1	2
HPAIV H5N1-W1	P	L	R	E	K	R	R	K	R	G	L	F
HPAIV H5N8-W1	P	L	R	E	K	R	R	K	R	G	L	F
SB-H5N1-W1	P	L	G	E	-	-	-	T	R	G	L	F
SB-H5N8-W1	P	L	G	E	-	-	-	T	R	G	L	F
SB-H5N1-W1 (IVPI kidney)	P	L	G	E	-	-	-	T	R	G	L	F

Fig. S4 Outcomes from intravenous pathogenicity index (IVPI) of H5N1-W1 and H5N8-W1 LPAIV.

A. Blood collected from nine (SB-H5N1-W1) and 10 (SB-H5N1-W1) chickens inoculated for an IVPI and then tested by HI against homologous wt HPAIV and de-engineered AIV antigens. **B.** Viral RNA detection in organs of one chicken which was found dead at 6 days post-inoculation with H5N1-W1, during the IVPI determination. **C.** Sequence comparison of the cleavage sites of the wild type (wt) HPAIV and de-engineered SB-H5N1-W1 and SB-H5N8-W1, as well as virus found in the kidney sample of the one chicken from **B**.
